# Superiority of Supervised Machine Learning on Reading Chest X-Rays in Intensive Care Units

**DOI:** 10.3389/fmed.2021.676277

**Published:** 2021-10-15

**Authors:** Kumiko Tanaka, Taka-aki Nakada, Nozomi Takahashi, Takahiro Dozono, Yuichiro Yoshimura, Hajime Yokota, Takuro Horikoshi, Toshiya Nakaguchi, Koichiro Shinozaki

**Affiliations:** ^1^Department of Emergency and Critical Care Medicine, Graduate School of Medicine, Chiba University, Chiba, Japan; ^2^Center for Frontier Medical Engineering, Chiba University, Chiba, Japan; ^3^Department of Diagnostic Radiology and Radiation Oncology, Chiba University Graduate School of Medicine, Chiba, Japan; ^4^Department of Emergency Medicine, Donald and Barbara Zucker School of Medicine at Hofstra/Northwell, Hempstead, NY, United States

**Keywords:** machine learning technique, chest radiographs, ICU, computer-aided detection, deep convolutional neural network, adaptive ensemble learning

## Abstract

**Purpose:** Portable chest radiographs are diagnostically indispensable in intensive care units (ICU). This study aimed to determine if the proposed machine learning technique increased in accuracy as the number of radiograph readings increased and if it was accurate in a clinical setting.

**Methods:** Two independent data sets of portable chest radiographs (*n* = 380, a single Japanese hospital; *n* = 1,720, The National Institution of Health [NIH] ChestX-ray8 dataset) were analyzed. Each data set was divided training data and study data. Images were classified as atelectasis, pleural effusion, pneumonia, or no emergency. DenseNet-121, as a pre-trained deep convolutional neural network was used and ensemble learning was performed on the best-performing algorithms. Diagnostic accuracy and processing time were compared to those of ICU physicians.

**Results:** In the single Japanese hospital data, the area under the curve (AUC) of diagnostic accuracy was 0.768. The area under the curve (AUC) of diagnostic accuracy significantly improved as the number of radiograph readings increased from 25 to 100% in the NIH data set. The AUC was higher than 0.9 for all categories toward the end of training with a large sample size. The time to complete 53 radiographs by machine learning was 70 times faster than the time taken by ICU physicians (9.66 s vs. 12 min). The diagnostic accuracy was higher by machine learning than by ICU physicians in most categories (atelectasis, AUC 0.744 vs. 0.555, *P* < 0.05; pleural effusion, 0.856 vs. 0.706, *P* < 0.01; pneumonia, 0.720 vs. 0.744, *P* = 0.88; no emergency, 0.751 vs. 0.698, *P* = 0.47).

**Conclusions:** We developed an automatic detection system for portable chest radiographs in ICU setting; its performance was superior and quite faster than ICU physicians.

## Introduction

Critically ill patients often have organ dysfunction and require frequent and intense monitoring. Portable chest radiography is key to assessing cardiopulmonary function in the intensive care unit (ICU), allowing clinicians to identify pathological findings such as pneumonia, pneumothorax, pleural effusion, and atelectasis (1–7). A review of a large number of portable chest radiographs with high accuracy is important for the improvement of ICU patient outcomes; however, this can be challenging, primarily due to a lack of manpower (8, 9). Machine learning technology is effective in analyzing a large amount of data, including image data (10–14). Therefore, this promising technique potentially supports interpretations of radiographs, which may improve quality of care and patient safety by reducing physician's workload in ICU.

Substantial investigations have documented computer-aided detection (CAD) systems for medical images (15–21). Advances in machine learning enhance the potential utility of ICU care. Among various medical images, chest radiographs have been the most investigated; however, insufficient accuracy limits its clinical use. In addition, investigations on chest radiographs in the ICU have not been well elucidated.

Therefore, we developed a new algorithm using supervised machine learning with two independent datasets. We hypothesized that the accuracy of our supervised machine learning technique would increase as the number of radiograph readings increased; we also hypothesized that the technique would accurately and quickly identify pathological findings from portable chest radiographs in a clinical setting.

## Methods

### Data Collection

#### We Collected Two Independent Data Set From Institutions in Different Regions

Data set 1 (a single Japanese center intensive care unit data): Consecutive portable chest radiographs (Sirius Starmobile tiara Airy; HITACHI, Tokyo, Japan) of a hospital based radiographic database for ICU patients who admitted between April 2017 and December 2018 were retrospectively extracted and used by the study team member from the ICU at Chiba University Hospital, Japan. This tertiary referral hospital ICU where approximately 1,800 patients admitted in a year had 22 beds and was utilized by patients following emergency room admission and after elective surgery, accounting for approximately 80% of ICU beds. Of 3,351 screened patients, we selected 380 chest radiographs, in which a single diagnosis could be made from one of the following categories: atelectasis, pneumonia, pleural effusion, and no emergency. The diagnosis was made on the basis of clinical signs, laboratory data, and other images, including computed tomography (CT) with a radiologist report and bedside ultrasound. Two board-certified ICU physician with the specialty of interventional radiology reviewed all radiographs and labeled each imaging with one of the diagnoses in a comprehensive and coherent manner. If there was any doubt in diagnosis, the radiograph was excluded from further analysis.

**Figure 1:**
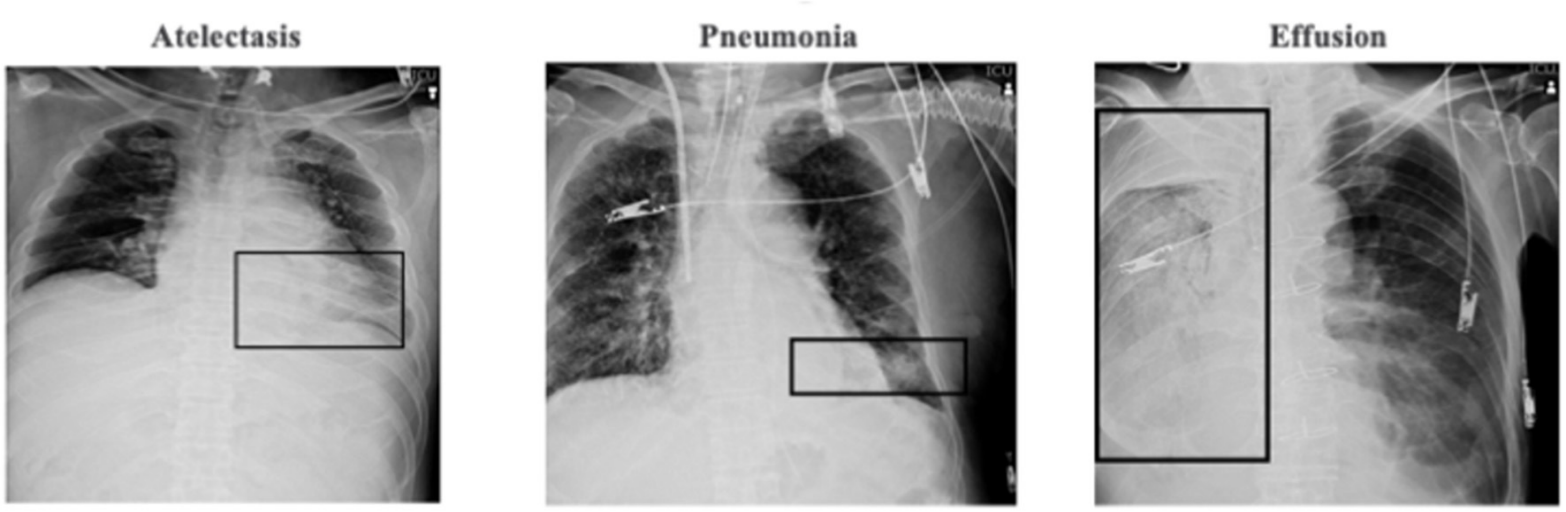
NIH repository test image sample.

Data set 2 (National Institute of Health [NIH] repository, US multi centers data): ChestX-ray8 dataset provided by the NIH clinical center, which contains 8,508 weak supervised multi-label methods to classify and locate the text-mined 14 common thorax diseases, mined from the text radiological reports via natural language processing techniques were used (Figure 1). Because the accuracy of this dataset is estimated to be >90%, we identified erroneous labels and cleaned up the images according to previous reports (22); we excluded images with magnification or those deemed to be of poor quality by three experts including two board-certified radiology physicians.

All clinical datasets were provided with DICOM format. The resolution of the original images in the single center ICU datasets sizes 2,430 pixels height and 1,994 pixels width and that of original images in the NIH datasets sizes 1,024 pixels height and 1,024 pixels width. To align them, all images in the single center ICU datasets are cropped and resized into 1,024 pixels height and 1,024 pixels width.

This study was approved by the Institutional Review Board of Chiba University Graduate School of Medicine (No. 2972; Feb, 2018) and was performed in accordance with the committee's guidelines.

### Two-Stage Classification Model

We used two different deep convolutional neural network architectures. Dense convolutional network 121 (DenseNet 121), which connects each layer to every other layer in a feed-forward fashion named DenseBlock. DenseNet121 has several compelling advantages and details are found in the supplemental digital content [see Appendix (Supplemental Digital content)].

The area under the curve (AUC) for atelectasis and pneumonia was particularly low at 0.574 and 0.499, respectively, in the four-class simultaneous classification of atelectasis, pneumonia, pleural effusion, and no abnormalities in DenseNets 121 conducted in a preliminary experiment. Therefore, we developed a two-stage classification method in which the combination of atelectasis and pleural effusion, and pneumonia and pleural effusion were once classified as the same class, and then separated them into each class in the second stage. The proposed network model of this study runs on Keras 2.1.5 with Python 3.4 on Ubuntu 16.04.4 LTS.

### Adaptive Ensemble Learning

In adaptive ensemble learning, an arbitrary number of models are selected from 10 models generated by iterative learning, and the average of the certainty output values of each model is calculated. For model selection, the optimum number and optimum combination were determined from 10 models. We found that it was optimal to select 4 out of 10 models in the proposed two-stage classification method. Using this adaptive ensemble learning, the average AUC improved to 0.672.

### Diagnostic Performance by Physicians vs. Machine Learning

To compare the accuracy and efficiency of the machine learning algorithm, five board-certified critical care physicians and three senior emergency residents voluntarily annotated images from the clinical samples which were dedicated data and part of the Chiba university hospital collected. To test physician's diagnostic accuracy, we developed a web-based software which was developed originally in our laboratory based on Java Script and can show a portable chest radiograph to a physician and the physician can input the diagnosis adjusting the dial of for categories (Not emergency, Pleural effusion, atelectasis, pneumonia) according to the physician's confidence from 0 to 100. We chose 53 chest radiographs which were randomly selected from Dataset 1. In a test, one physician successively reviewed 53 chest radiographs one by one using the developed software which automatically record the duration to diagnose a single portable chest radiograph (Supplementary Figure 1, Supplementary Material). The diagnostic accuracy calculated by AUC of receiver operator characteristic (ROC) analysis and the time for completing the images were compared with those obtained using the machine algorithm.

### Outcome Measures and Statistical Analysis

The sensitivity and specificity for the ability of the CNN to classify atelectasis, pneumonia, pleural effusion, and no emergency were calculated. The ROCs were plotted by varying the operating threshold. For the ROC curves, standard error, 95% confidence intervals, and comparisons between AUCs were made using a non-parametric approach. The adjusted Wald method was used to determine 95% confidence intervals for sensitivity and specificity. *P*-values < 0.05 were considered to be statistically significant. All statistical analyses were performed using R^®^ (ROCR, version 3.2.4) and PRISM^®^ version 7 (GraphPad Software, Inc., La Jolla, CA, USA).

## Results

Table 1 depicts the process of our algorithm development using Data set 1(a single Japanese center intensive care unit data). As shown in the table, our two-stage classification model improved its performance as compared to that of a four-class simultaneous classification model.

**Table 1:** Accuracy comparison of four algorithms.

**Comparison of AUC**	**Atelectasis**	**Pneumonia**	**Effusion**	**Not emergency**	**Four classes** **average**
Four-class simultaneous classification	0.574	0.499	0.700	0.625	0.600
Two-stage classification 1	0.605	0.551	0.599	0.518	0.568
Two-stage classification 2	0.672	0.686	0.676	0.561	0.649
Two-stage classification 2 and adaptive ensemble learning	0.711	0.698	0.718	0.634	0.690

*AUC, area under the curve*.

**Figure 2:**
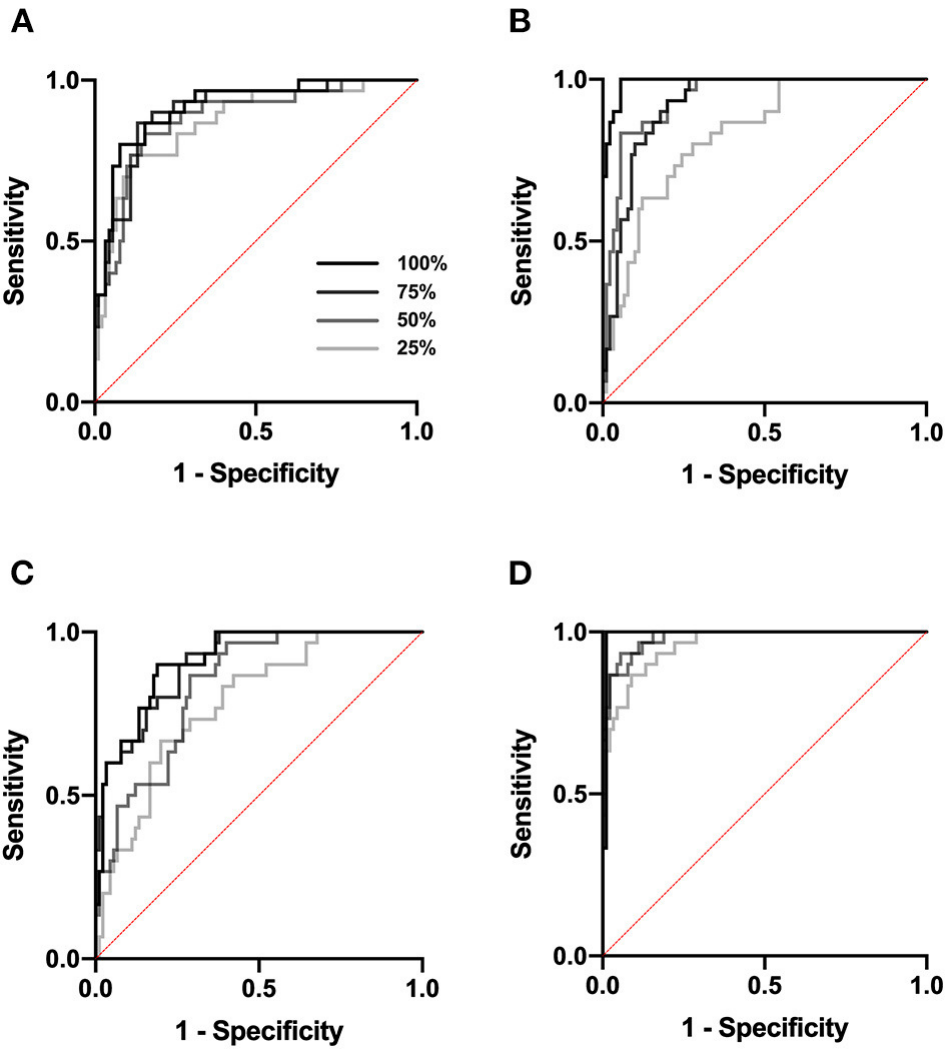
ROC curve. ROC analysis was performed at a sample size of 25, 50, 75, and 100%. **(A)** ROC analysis for atelectasis. **(B)** for pleural effusion. **(C)** for pneumonia. **(D)** for no emergency.

**Figure 3:**
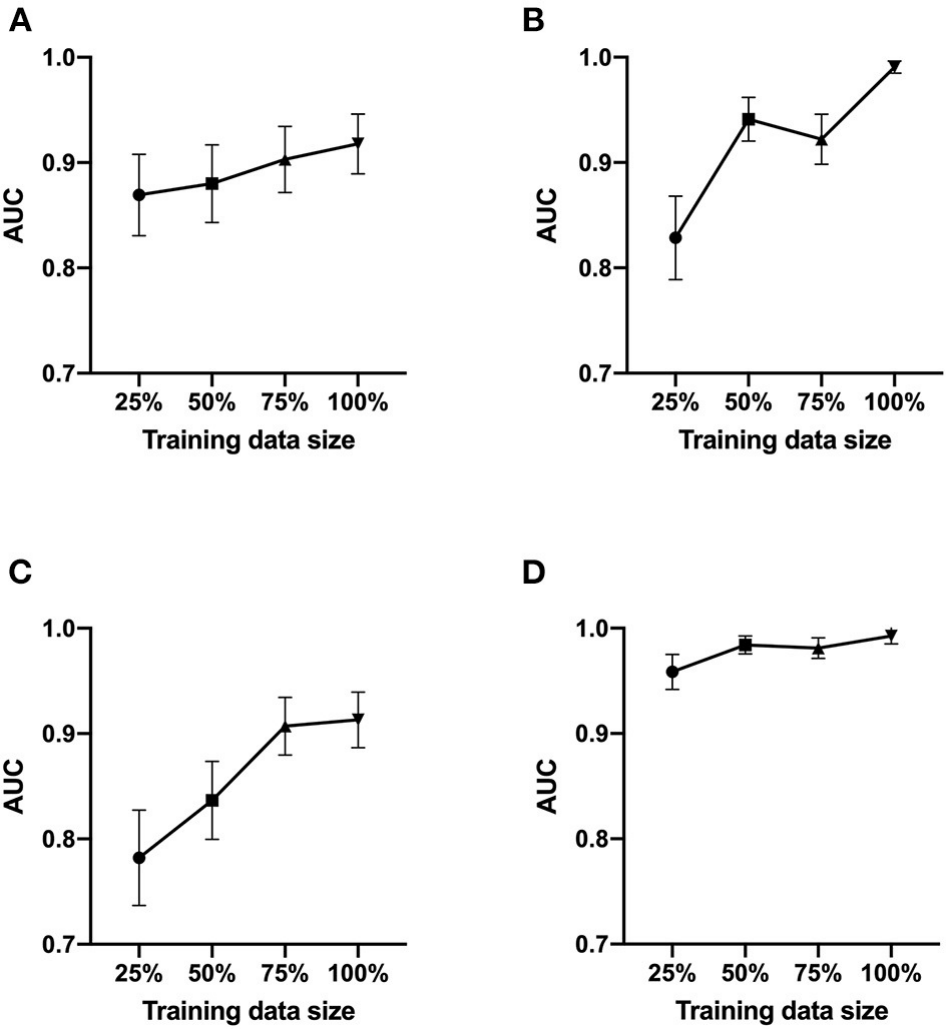
AUC values as a function of the sample size. The AUC of ROC analysis was shown at a sample size of 25, 50, 75, and 100%. **(A)** for atelectasis. **(B)** for pleural effusion. **(C)** for pneumonia. **(D)** for no emergency.

We next tested Data set 2 (NIH repository data, US multi center data, which is a larger and independent data set from different region, includes 1,120 for training, 120 for validation and 480 for test). In atelectasis, the AUC increased as the number of training data points increased, with a significant difference between 25 (280 images) and 100% (1,120 images) of the samples (*P* < 0.05) (Figure 2A). In pleural effusion, the AUC increased with an increase in the amount of data from 25 to 50% (*P* < 0.01) and from 75 to 100% of the samples (*P* < 0.01) (Figure 2B). In pneumonia, the AUC increased from 25 to 50% (*P* < 0.05) and 75% (*P* < 0.01) of the samples with an increase in the number of data points, respectively (Figure 2C). For no emergency, the AUC showed an increase in the number of data points from 25 to 50% (*P* < 0.05) and 25 to 100% of the samples (*P* < 0.05) (Figure 2D), with an increase in the number of data points. Figures 3A–D depicts the trend of AUC improvement with the increase in sample numbers.

The average time for ICU physicians to complete a review of 53 chest radiographs was 12 min (median, interquartile range 9–14). The algorithm finished the same dataset within 9.66 s. The diagnostic accuracy represented by AUC was higher by machine learning compared to those physicians in all categories: atelectasis, AUC 0.744 (95%CI 0.583–0.904) vs. 0.557 (95%CI 0.507–0.636), *P* = 0.030; pleural effusion, AUC 0.856 (95%CI 0.754–0.958) vs. 0.706 (95%CI 0.657–0.752), *P* = 0.007; pneumonia, AUC 0.702 (95%CI 0.571–0.869) vs. 0.744 (95%CI 0.643–0.829), *P* = 0.881; no emergency, AUC 0.751 (95%CI 0.597–0.906) vs. 0.698 (95%CI 0.615–0.792), *P* = 0.476).

## Discussion

In the present study, we developed a novel automatic detection system to aid ICU physicians in identifying pathological findings from portable chest radiographs. In the case of atelectasis and pleural effusion, the system performed extremely well compared to board-certified ICU physicians. Another advantage that should be highlighted is that the supervised machine learning technology had an extremely fast diagnostic time. The system could interpret one image per 0.18 s. Since the proposed model can run on the general deep learning framework, it can be adapted to widely other environments.

In this study, we validated that the diagnostic accuracy improved as the sample size increased with a large sample size (NIH repository data). The accuracy of this dataset is considered to be >90%, and so it would be sufficient to test our hypothesis. However, because of the uncertainty of the diagnosis randomly lurk under a cloak of the provided chest radiographs, we used our own in-hospital dataset to develop the machine learning algorithm, which is key to improving the accuracy of our system. We developed a novel two-stage classification method from this dataset. Incorporating machine learning into diagnostic imaging could lead to rapid therapeutic interventions by determining image results quickly with fewer errors. As for the cost, it is unknown since the business has not yet been developed.

It is important to aim for higher sensitivity to rule out acute pathology. Sensitivity is referred to as recall in the machine learning field. The sensitivity of our system for acute pathology was 0.846 for atelectasis, 0.846 for pneumonia, and 1.00 for pleural effusion. On the basis of our findings, the clinical application of this system is wide; the technology allows for reducing the risk of misdiagnosis and saving physician's efforts. The system requires true diagnosis; correct labels of radiographs need to be provided by clinicians. However, our data indicate that, after a period of training, once the system has taken a sufficient sample size, an end-user will experience high satisfaction in the diagnoses; the diagnoses would be consistent with their own at high accuracy (AUC >0.9) and at an extremely fast processing time.

While detecting pneumonia, machine learning had a poorer performance compared to the other abnormal findings. Poor performance in machine learning is either due to overfitting or underfitting of the data. Overfitting occurs when the trained model does not generalize well to unseen cases but fits the training data well. Assessment of the training curves can be used to evaluate the possibility of overfitting. In our study setting, it was apparent that the data loss on the validation data was much greater than that on the training datasets, which suggested the possibility of overfitting. The trend becomes more prominent when the training sample size is small. This is one of the main limitations of this study.

Since the present study developed algorithms with high predictive value, the future direction of research would be clinical application. The COVID-19 pandemic would require the speedy X-ray diagnosis system with high processing capability which detect the severe pneumonia in the shortage of the specialist physician (23–26). This system may be helpful for the screening of these patients with pulmonary opacity.

The study had other limitations that should be addressed. First, the number of learning images needed to be reduced by removing duplicate labels from the dataset. For example, for pneumonia, the ChestX-ray14 database had 1,107 pneumonia images; however, those included other labels such as pneumonia/atelectasis, pneumonia/pleural effusion/, and pneumonia/mass. We chose 300 images that were solely labeled as pneumonia to avoid the double count of diagnosis. It is plausible that the system performance decreased because the number of learning images was limited in pneumonia. Second, the resolution of image samples was lower than that of normal X-ray images. In our hospital, physicians usually use images with a resolution higher than 2,000 pixels; however, the images needed to be resized to 1,024 pixels in the training and validation datasets. Further studies are warranted to test the effect of image resolutions on diagnostic accuracy by comparing physicians and machine learning techniques. Third, this study lacked the topographic diagnoses within the chest X-rays (pathology by lobes, or by segments), and other pathologies or information that can be read in the radiographs, such as positioning of devices. However, this would not be a problem except for the increase in the amount of information that would have to be contributed to the learning system or for the addition of other layers of neurons to the model. Furthermore, this study did not have the data of the baseline demographic and clinical characteristics of participants including patients age, sex, BMI, diagnosis, and SOFA score. However, the machine learning without patient's characteristics would be versatile and increase the ability of accuracy form the images with limited information. Finally, this study has several limitations including sources of potential bias, statistical uncertainty, the limited number of cases, the number of investigators from the same geographical area, and generalizability in another environment (different types of patients, races, pathologies). Statistical uncertainty is important since the sample size calculation was not performed in this study. Since we developed new prediction algorithms using machine learning approaches and could not speculate a proper sample size, we did perform sample size estimation and analyzed available data. However, the strength of this study is comparison of the software performance with human chest x-ray readers. Expansive study, which is statistically built using this study results and add new images, in particular from different geographical regions to improve performances and generalizability of the model with multicenter even international collaborations, would be desirable.

## Conclusions

We developed an automatic detection system for portable chest radiographs of ICU patients using two-stage classification method which performed superior to board-certified ICU physicians in the case of atelectasis and pleural effusion. The diagnostic accuracy improved as the sample size increased. The diagnostic time of the machine learning system was significantly shorter than that of physicians.

## Data Availability Statement

The raw data supporting the conclusions of this article will be made available by the authors, without undue reservation.

## Ethics Statement

The studies involving human participants were reviewed and approved by Institutional Review Board of Chiba University Graduate School of Medicine. The Ethics Committee waived the requirement of written informed consent for participation.

## Author Contributions

KT and TNakad designed the conception of the study. KT, TD, and TNakag performed acquisition of data. KT, NT, TD, and TNakag analyzed statistics. KT and KS drafted the manuscript. All authors made interpretations of data, added intellectual content of revisions to the paper, and gave final approval of the version to be published.

## Funding

Research reported in this publication was supported by an institutional internal funding.

## Conflict of Interest

KS has a patent right of metabolic measurements in critically ill patients and grant/research supports from Nihon Kohden Corp. This does not alter the authors' adherence to all the journal's policies on sharing data and materials. TNakad, KT, TD, YY, and TNakag are the inventor of the patent. TNakad is the CEO of Smart119 Inc. and owns the stock. The remaining authors declare that the research was conducted in the absence of any commercial or financial relationships that could be construed as a potential conflict of interest.

## Publisher's Note

All claims expressed in this article are solely those of the authors and do not necessarily represent those of their affiliated organizations, or those of the publisher, the editors and the reviewers. Any product that may be evaluated in this article, or claim that may be made by its manufacturer, is not guaranteed or endorsed by the publisher.
