# Supplementary material for: Superiority of Supervised Machine Learning on Reading Chest X-Rays in Intensive Care Units
Source: Front Med (Lausanne). 2021 Oct 15;8:676277. doi: 10.3389/fmed.2021.676277 (PMC8554032; doi:10.3389/fmed.2021.676277)
Supplement: Supplementary file 1 [file Presentation_1.pdf]

Supplementary Material

Superiority of Supervised Machine Learning on Reading Chest X-rays in Intensive Care Units

Kumiko Tanaka M.D.[1]; Taka-aki Nakada M.D., Ph.D.[1]; Nozomi Takahashi M.D, Ph.D.[1]; Takahiro Dozono BE[3], Yuichiro Yoshimura PhD[3], Hajime Yokota MD, PhD[4], Takuro Horikoshi MD, PhD[4], Toshiya Nakaguchi PhD[3] and Koichiro Shinozaki M.D, Ph.D.[1,2]

[1] Department of Emergency and Critical Care Medicine, Graduate School of Medicine, Chiba University, Chiba, Japan

[2] Department of Emergency Medicine, Donald and Barbara Zucker School of Medicine at Hofstra/Northwell, Hempstead, NY, USA

[3] Center for Frontier Medical Engineering, Chiba University, Chiba, Japan

[4] Chiba University Graduate School of Medicine, Department of Diagnostic Radiology and Radiation Oncology, Chiba, Japan

*** Correspondence:**
Taka-aki Nakada

Chiba University Graduate School of Medicine, Department of Emergency and Critical Care Medicine, 1-8-1 Inohana, Chuo, Chiba 260-8677, Japan

Phone: +81-43-226-2372, Fax: +81-43-226-2371,

Email: taka.nakada@chiba-u.jp

Methods

Data Collection

Data set 1 (A single Japanese center ICU data): Consecutive portable chest radiographs (Sirius Starmobile tiara airy; HITACHI, Tokyo, Japan) were retrospectively collected in ICU at Chiba university hospital, Japan, from April 2017 to December 2018. Of 3351 patients screened, we selected 380 chest radiographs, in which a sole diagnosis was able to be made from one of the followings: atelectasis, pneumonia, pleural effusion, and no emergency. The diagnosis was made on the basis of clinical signs, laboratory data, and other images including computed tomography (CT) with a radiologist report and bedside ultrasound. The images were randomly assigned into training and test data where the number of test data in each class is 20.

Data set 2 (National Institute of Health (NIH) repository, US multi centers data): ChestX-ray8 dataset provided by NIH clinical center, which contains 8508 weak supervised multi-label methods to classify and locate the text-mined 14 diseases, mined from the text radiological reports via natural language processing techniques were used. Since the accuracy of this dataset is estimated to be >90%, we identified erroneous labels and cleaned up the images according to previous reports [1], and excluded images with magnification or poor quality by three experts including two board-certified radiology physicians. The images were randomly assigned into the training and test data where the number of test data in each class is 120.

Two-stages classification model

We used two different deep convolutional neural network architecture, DenseNet121. Dense Convolutional Network 121 (DenseNet 121), which connects each layer to every other layer in a feed-forward fashion named DenseBlock. DenseNet121 have several compelling advantages: they reduce the vanishing-gradient problem, intensify feature propagation, encourage feature reuse, and substantially reduce the number of parameters.

All CNN layers were fine-tuned using stochastic gradient descent with a global learning rate of 0.001, a batch size of 16 and 500 epochs. Each image was resized to 1024*1024 pixels. Augmentation technique was horizontal flip, rotation ±7 degrees. Due to the difference in brightness between the single center ICU data and NIH, gamma correction=0.5 for the single center ICU images was applied.

We used a computer with Intel Xeon E5-s2640 2.4Ghz to build the teaching data set. The distributions of diagnoses are indicated based on the image features, and some difficult radiography images were proved by the computer tomography images.

The AUCs for atelectasis and pneumonia were particularly low at 0.574 and 0.499, respectively, in the four-class simultaneous classification of atelectasis, pneumonia, pleural effusion, and no abnormalities in DenseNet 121 conducted as a preliminary experiment. In addition, many images

were misclassified as pleural effusion in both atelectasis and pneumonia classes. From the above results, it is considered necessary to improve the classification of pleural effusion in order to accurately classify atelectasis and pneumonia at the same time. Therefore, we examined the following two types of two-stage classification methods in which the combination of atelectasis and pleural effusion and pneumonia and pleural effusion were once classified as the same class, and then reclassified between the classes in the same class.

Case 1: A method of fusing pneumonia and pleural effusion into one class, classifying them into three classes of atelectasis and no abnormalities, and then classifying them into two classes of pneumonia and pleural effusion in the second stage.

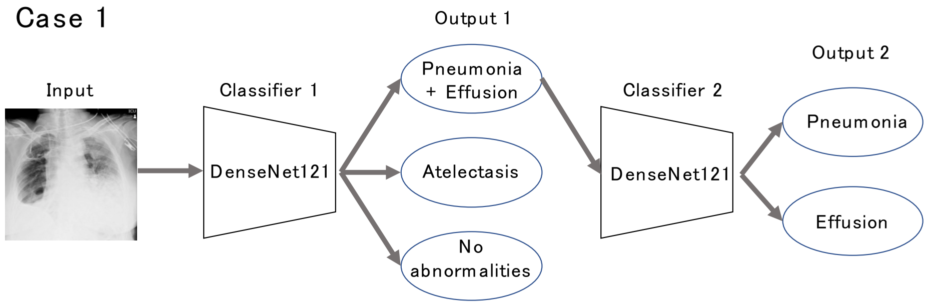

Case 2: A method in which atelectasis and pleural effusion are fused into one class, classified into three classes of pneumonia and no abnormality, and then classified into two classes of atelectasis and pleural effusion in the second stage classification.

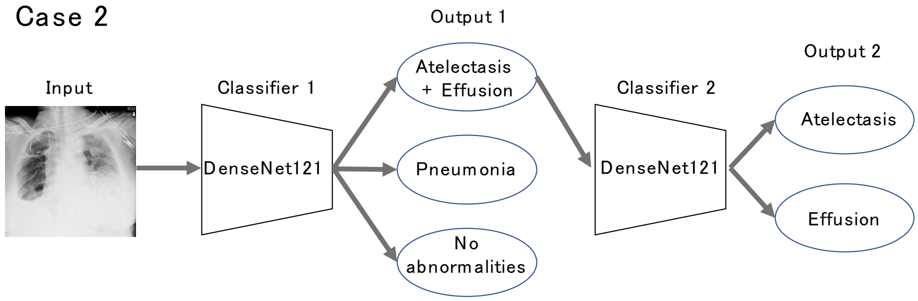

The specific flow will be explained using the case 1. The input images are classified into three classes, pneumonia + pleural effusion, atelectasis, and pneumonia in the first stage. If it is judged as pneumonia + pleural effusion class in the first stage, the image is classified into two classes, pneumonia and pleural effusion in the second stage. Then, among the classification probabilities for all classes, the class with the highest classification probability is used as the final output result. The number of training images for each class was prepared for each stage. In the case 1, 35 pneumonia and 139 pleural effusion were used as one class, and the other two classes were set as 69 each. Similarly, in the case 2, 39 atelectasis and 139 pleural effusion were used for one class, and the other two classes were used for 77 each. In the second stage, new images were added to the images used in the first stage, and pneumonia and pleural effusion were 77 each in case 1, and atelectasis and pleural effusion were 69 each in case 2. We compared the learning performance of case 1 and case 2. In-hospital data was used for the test, and the area under curve (AUC) value of the receiver operating curve (ROC) was calculated as the evaluation index. As a result, the 4-class average AUC of case 1

was 0.569, and the average AUC of case 2 was 0.649. Therefore, the case 2 model is adopted in this study.

Adaptive ensemble learning

When the reproducibility of the training model was evaluated, it was confirmed that there was a significant difference in accuracy between the models trained under the same conditions. Therefore, we hypothesized that combining models with different output results would complement each other's misclassification, and proposed adaptive ensemble learning that arbitrarily combines the outputs of 10 models that were repeatedly trained under the same conditions. This is an improved method of conventional ensemble learning [2]. In adaptive ensemble learning, an arbitrary number of models are selected from the 10 models generated by iterative learning, and the average of the certainty output values of each model is calculated. For model selection, the optimum number and optimum combination were determined from 10 models. Here we explain the detail flow of our adaptive ensemble learning. First, the average classification probability of each class is calculated using the classification probabilities of the three classes for the input image in the first stage of each model to be combined. Next, if the highest class among the calculated average classification probabilities is "atelectasis or pleural effusion", the average classification probability of atelectasis and pleural effusion is calculated in the second stage. In the first and second stages, the class with the highest average classification probability is the result of ensemble learning. Comparing the results of one model alone with the results of ensemble learning, it was confirmed that the accuracy was improved by ensemble learning, suggesting its effectiveness. However, when examining a combination of 10 models, it takes a lot of time because it is necessary to calculate all the results in 1013 ways to select models. Therefore, we used the correlation-based search, which uses the correlation values of each model for the purpose of reducing the number of searches. By using the correlation value, we hypothesized that it is possible to search for combinations that can efficiently complement each other's misclassifications. When the correlation-based search was applied to model search in ensemble learning of image classification models using 4-class simultaneous classification and 2-step classification, the number of searches was reduced from 1013 to 30 with the similar performance as the Blute-force search. As a result of this study, it was found that it is optimal to select 4 out of 10 models in the proposed two-stages classification method. By using this adaptive ensemble learning, the average AUC improved to 0.672.

Diagnostic performance by physicians vs. machine learning

To compare to the machine learning algorithm, 5 board-certified emergency physicians and 3 senior emergency residents voluntarily annotated images from the clinical samples. All physicians individually reviewed 53 chest radiograph and recorded labels for each image. Physicians were blinded from both clinical diagnosis and image labels. The diagnostic accuracy calculated by AUC of ROC analysis and the time for completing the images were compared those by machine algorithm.

sFigure 1. Physician's test image

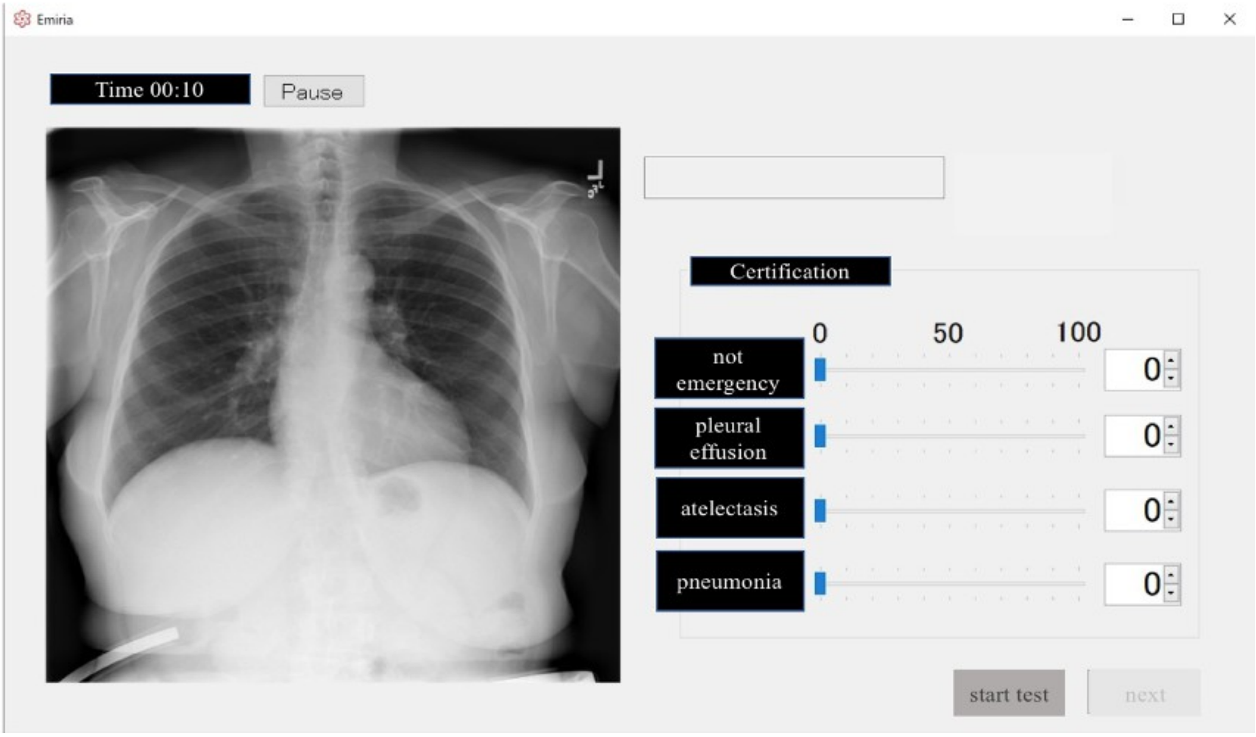

sFigure 2. ROC analysis

Table Area under the receiver operating characteristic curve.

			AUC(95%CI)		sensitivity(95%CI)		specificity(95%CI)	
	**Not emergency**							
3	Not.emergency1	1	0.819	(0.683 - 0.954)	0.769	(0.462 - 0.950)	0.897	(0.758 - 0.971)
9	Not.emergency2	5	0.684	(0.525 - 0.843)	0.538	(0.251 - 0.808)	0.868	(0.719 - 0.956)
9	Not.emergency3	3	0.792	(0.655 - 0.928)	0.769	(0.462 - 0.950)	0.821	(0.665 - 0.925)
9	Not.emergency4	4	0.791	(0.643 - 0.939)	0.692	(0.615 - 0.386)	0.846	(0.923 - 0.695)
9	Not.emergency5	6	0.667	(0.512 - 0.822)	0.538	(0.251 - 0.808)	0.795	(0.635 - 0.907)
3	Not.emergency6	7	0.598	(0.447 - 0.748)	0.385	(0.139 - 0.684)	0.872	(0.726 - 0.957)
18	Not.emergency7	8	0.474	(0.439 - 0.509)	1.000	(0.753 - NaN)	0.000	(NaN - 0.090)
4	Not.emergency8	2	0.758	(0.614 - 0.903)	0.615	(0.316 - 0.861)	0.923	(0.791 - 0.984)
	**Average**		**0.698**	**(0.615 - 0.792)**	0.654		0.857	
	Results of the system		0.751	(0.597 - 0.906)	0.615	(0.316 - 0.861)	0.821	(0.665 - 0.925)
	P-Value		0.476					
	**Effusion**							
3	Effusion1	6	0.694	(0.543 - 0.845)	0.846	(0.546 - 0.981)	0.590	(0.421 - 0.744)
9	Effusion2	3	0.738	(0.600 - 0.875)	0.923	(0.640 - 0.998)	0.564	(0.396 - 0.722)
9	Effusion3	2	0.757	(0.606 - 0.909)	0.692	(0.386 - 0.909)	0.795	(0.635 - 0.907)
9	Effusion4	7	0.645	(0.475 - 0.815)	0.462	(0.192 - 0.749)	0.872	(0.726 - 0.957)
9	Effusion5	5	0.711	(0.561 - 0.861)	0.615	(0.316 - 0.861)	0.821	(0.665 - 0.925)
3	Effusion6	4	0.728	(0.549 - 0.906)	0.615	(0.316 - 0.861)	0.897	(0.758 - 0.971)
18	Effusion7	8	0.585	(0.419 - 0.750)	0.308	(0.091 - 0.614)	0.923	(0.791 - 0.984)
4	Effusion8	1	0.792	(0.653 - 0.931)	0.846	(0.769 - 0.546)	0.692	(0.769 - 0.524)
	**Average**		**0.706**	**(0.657 - 0.752)**	0.654		0.808	
	Results of the system		0.856	(0.754 - 0.958)	1.000	(0.753 - 1.000)	0.667	(0.498 - 0.809)
	P-Value		**0.007**					
	**Atelectasis**							
3	Atelectasis1	8	0.462	(0.312 - 0.611)	0.308	(0.091 - 0.614)	0.718	(0.551 - 0.850)
9	Atelectasis2	4	0.544	(0.346 - 0.742)	0.538	(0.251 - 0.808)	0.718	(0.551 - 0.850)
9	Atelectasis3	2	0.659	(0.497 - 0.821)	0.538	(0.251 - 0.808)	0.769	(0.607 - 0.889)
9	Atelectasis4	7	0.501	(0.335 - 0.667)	0.462	(0.192 - 0.749)	0.615	(0.446 - 0.766)
9	Atelectasis5	3	0.568	(0.422 - 0.714)	0.308	(0.091 - 0.614)	0.846	(0.695 - 0.941)
3	Atelectasis6	6	0.526	(0.370 - 0.681)	0.385	(0.139 - 0.684)	0.692	(0.524 - 0.830)
18	Atelectasis7	5	0.533	(0.331 - 0.734)	0.308	(0.091 - 0.614)	0.897	(0.758 - 0.971)
4	Atelectasis8	1	0.665	(0.513 - 0.817)	0.462	(0.192 - 0.749)	0.897	(0.758 - 0.971)
	**Average**		**0.557**	**(0.507 - 0.636)**	0.423		0.744	
	Results of the system		0.744	(0.583 - 0.904)	0.846	(0.546 - 0.981)	0.667	(0.498 - 0.809)
	P-Value		**0.030**					
	**Pneumonia**							
3	Pneumonia1	7	0.628	(0.449 - 0.808)	0.615	(0.538 - 0.316)	0.641	(0.718 - 0.472)
9	Pneumonia2	6	0.689	(0.491 - 0.888)	0.692	(0.386 - 0.909)	0.821	(0.665 - 0.925)
9	Pneumonia3	3	0.802	(0.660 - 0.943)	0.692	(0.386 - 0.909)	0.897	(0.758 - 0.971)
9	Pneumonia4	4	0.789	(0.640 - 0.938)	0.769	(0.462 - 0.950)	0.744	(0.579 - 0.870)
9	Pneumonia5	5	0.751	(0.602 - 0.901)	0.615	(0.316 - 0.861)	0.897	(0.758 - 0.971)
3	Pneumonia6	8	0.600	(0.453 - 0.746)	0.308	(0.091 - 0.614)	0.923	(0.791 - 0.984)
18	Pneumonia7	1	0.851	(0.753 - 0.949)	1.000	(0.753 - NaN)	0.641	(0.472 - 0.788)
4	Pneumonia8	2	0.838	(0.701 - 0.975)	0.769	(0.462 - 0.950)	0.846	(0.695 - 0.941)
	**Average**		**0.744**	**(0.643 - 0.829)**	0.692		0.833	
	Results of the system		0.720	(0.571 - 0.869)	0.846	(0.546 - 0.981)	0.564	(0.396 - 0.722)
	P-Value		0.881					

AUC: area under the curve.
CI:Confidence interval
P-Value: DeLong's test for two ROC curves.

References

1. Xiaosong Wang YP LL, Zhiyong Lu, Mohammadhadi Bagheri, Ronald M. Summers: ChestX-ray8: Hospital-scale Chest X-ray Database and Benchmarks on Weakly-Supervised Classification and Localization of Common Thorax Diseases. *IEEE CVPR* (2017) 2097-2106.

2. Rajaraman S, Sornapudi S, Kohli M, Antani S: Assessment of an ensemble of machine learning models toward abnormality detection in chest radiographs. *Annu Int Conf IEEE Eng Med Biol Soc* (2019) 3689-3692.
